# Effect of the SARS-CoV-2 pandemic on planned and emergency hernia repair in Sweden: a register-based study

**DOI:** 10.1007/s10029-023-02828-6

**Published:** 2023-07-07

**Authors:** Fathalla Ali, Gabriel Sandblom, Blend Fathalla, Göran Wallin

**Affiliations:** 1 Faculty of Medicine and Health, Department of Surgery, Örebro University, 70185 Örebro, Sweden; 2 Department of Surgery, Karlskoga Hospital, 69144 Karlskoga, Sweden; 3 Department of Clinical Science and Education Södersjukhuset, Karolinska Institute, Stockholm, Sweden; 4 Emergency Department, Södersjukhuset, Stockholm, Sweden

**Keywords:** Delayed hernia repair, Acute hernia repair, COVID-19 pandemic, Planned hernia repair, Delayed surgery, Postponed hernia repair

## Abstract

**Purpose:**

The COVID-19 has had a profound impact on the health care delivery in Sweden, including deprioritization of benign surgeries during the COVID-19 pandemic. The aim of this study was to assess the effect of COVID-19 pandemic on emergency and planned hernia repair in Sweden.

**Methods:**

Data on hernia repairs from January 2016 to December 2021 were retrieved from the Swedish Patient Register using procedural codes. Two groups were formed: COVID-19 group (January 2020–December 2021) and control group (January 2016–December 2019). Demographic data on mean age, gender, and type of hernia were collected.

**Results:**

This study showed a weak negative correlation between the number of elective hernia repairs performed each month during the pandemic and the number of emergency repairs carried out during the following 3 months for inguinal hernia repair (*p* = 0.114) and incisional hernia repair (*p* = 0.193), whereas there was no correlation for femoral or umbilical hernia repairs.

**Conclusion:**

The COVID-19 pandemic had a great impact on planned hernia surgeries in Sweden, but our hypothesis that postponing planned repairs would increase the risk of emergency events was not supported.

## Introduction

The coronavirus disease 2019 (COVID-19) pandemic has had a great effect on the health care system in Sweden [1]. Sweden’s approach to the COVID problem differed from that of many other Western countries in that the authorities did not impose a nationwide lockdown [2]. The first confirmed case of COVID-19 in Sweden was reported in Jönköping on January 24, 2020 [3], and the first patient death was reported in Stockholm on March 11, 2020 [4]. The first outbreak in Sweden occurred in Stockholm after the school winter holiday between February 24 and March 1, after which many families returned from skiing in the Alps [5]. This coincided with the outbreaks in Lombardy in northern Italy and Austria. Thereafter, the disease rapidly spread from Stockholm, and the first wave of the pandemic in Sweden was underway [5].

COVID-19 had a far-reaching impact on the health care system, forcing a widespread reallocation of resources and medical personnel [6–8]. This resulted in the postponement of many operations for benign conditions [9–11]. It is estimated that worldwide, more than 2 million operations were delayed per week during the first wave of the pandemic, the majority of which were planned [8, 12]. It is still not clear, however, whether the postponed planned surgeries led to increased emergency events.

This study aimed to explore the impact of the COVID-19 pandemic on hernia surgeries in Sweden and to determine whether fewer planned hernia surgeries resulted in an increase in emergency cases. We hypothesized that a lower number of elective hernia repairs during a particular month during the pandemic in Sweden was associated with an increase in the number of emergency repairs during the following 3 months.

## Materials and methods

The period between January 2020 and December 2021 was considered the COVID-19 period in this study. The numbers of hernia repairs during the pre-pandemic years from January 2016 to December 2019 were used for comparison. Healthcare in Sweden is decentralized, with each region making their own prioritizations. There was no standard routine on a national level for the management of elective surgery during the study period.

Data on hernia repairs were retrieved from the Swedish National Patient Register. The Swedish National Patient Register is a register covering all inpatient care as well as outpatient visits in Sweden [13]. All patients are registered with their personal identity numbers, ICD codes, and surgical procedure code in the register. There is also information on whether the admission was planned or unplanned. Since 1987, the National Patient Register includes all in-patient care in and since 2001 outpatient visits as well. The validity of the NPR has been shown to be high for the diagnoses relevant for the present study [14].

The surgical procedures were identified using the following ICD codes: K40 (inguinal hernia), K41 (femoral hernia), K42 (umbilical hernia), and K43.0–K43.5 (incisional hernia). Planned and unplanned admissions were included. In case the admission was unplanned, the hernia was assumed to be incarcerated or strangulated and the repair was assumed to have been an emergency procedure. There were no specific inclusion or exclusion criteria. Inpatient as well as outpatient procedures were included.

The sum of subsequent three months was chosen for assessing the emergency hernia repairs during COVID-19 pandemic. We chose the cut-off limit of three months to cover the period when the risk of incarceration in case of non-operative management is greatest. In a previous study, half of the patients who presented with incarceration within a year after diagnosis of an incisional hernias developed the incarceration the first three months after a primary decision of non-operative management [15].

Data on new cases of COVID-19 were retrieved from the Swedish Department of Public Health [16]. By law, all cases of confirmed COVID-19 were reported to the department. We also retrieved data on patients admitted to an intensive care unit (ICU) with COVID-19.

**Fig. 1:**
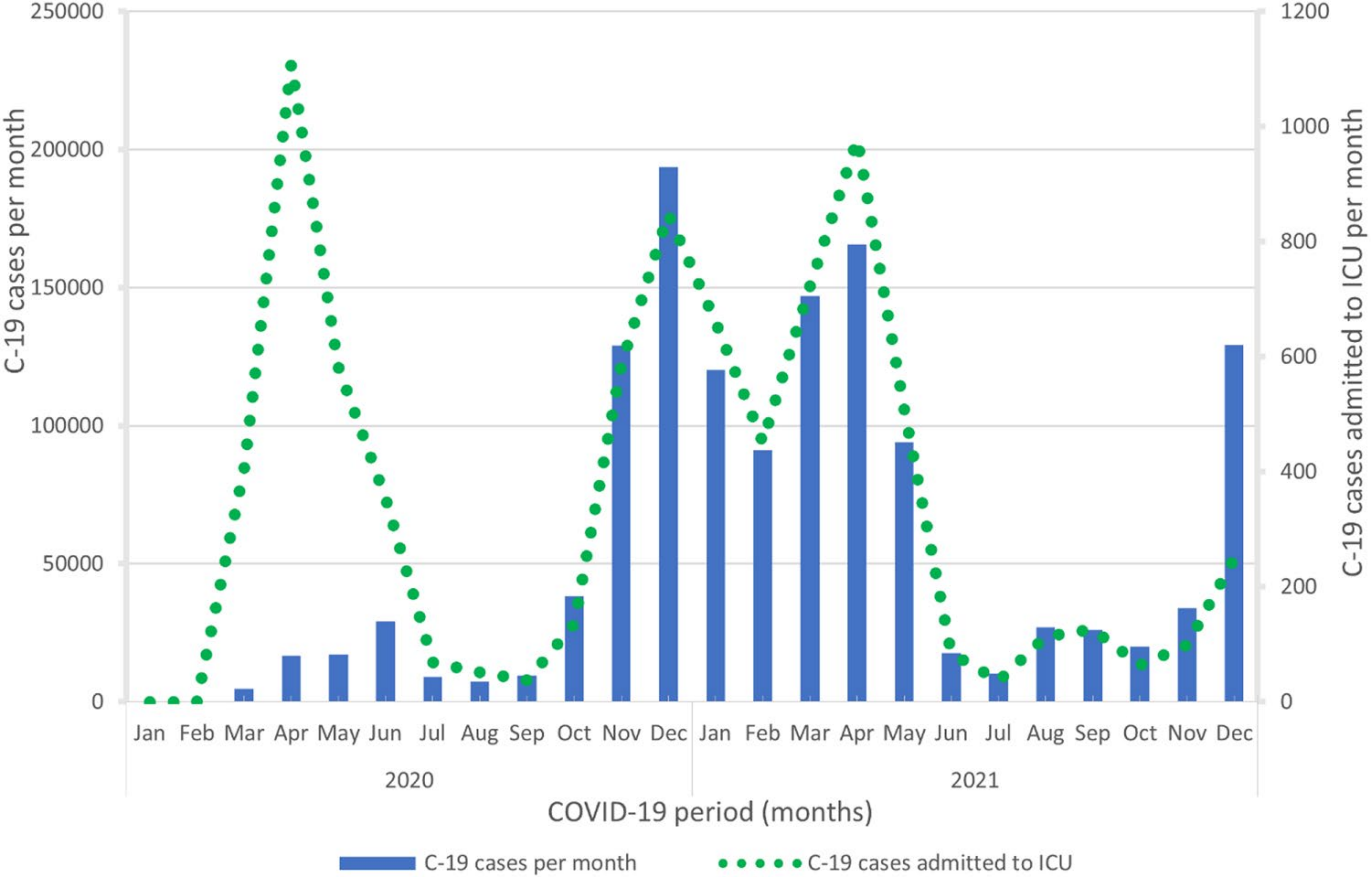
Number of patients with a COVID-19 diagnosis (left y-axis) and number of COVID-19 patients admitted to the ICU in Sweden each month in 2020–2021 (right y-axis)

COVID-19 cases and those admitted to the ICU during the COVID study period followed the same pattern due to the large number of severely ill patients between March 1, 2020 and May 31, 2021, which showed the greatest burden to the system (Fig. 1).

### Statistical analyses

Statistical analyses were performed using SPSS (Statistical Package for the Social Sciences for Windows, version 28.0, Armonk, NY, USA; IBM Corp.). To test whether a lower number of elective hernia repairs resulted in an increase in emergency events, we looked for an inverse relationship between the number of planned repairs during each month of the COVID period and the number of emergency repairs during the following three months. Relationships were tested with Pearson’s correlation test. A *p* value < 0.05 was considered statistically significant.

## Results

The number of COVID-19 cases and the number of people admitted to the ICU are presented in Fig. 1. Admission to the ICU reflects those periods with the most intensive burden on health care.

**Fig. 2:**
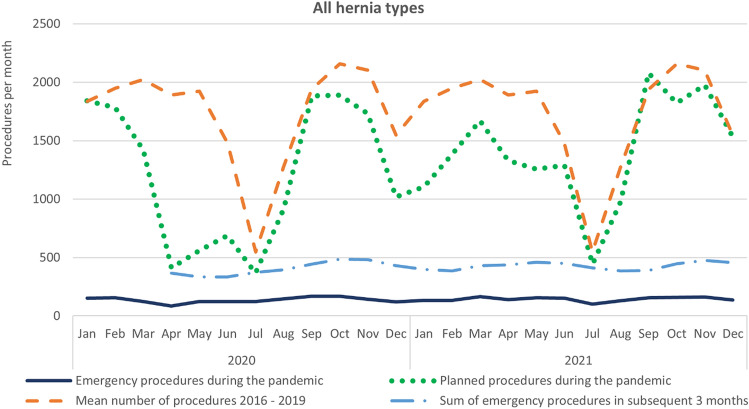
Mean numbers of hernia repairs each month. The COVID study period was 2020–2021. For comparison, mean figures from the pre-pandemic period 2016–2019 were used

**Fig. 3:**
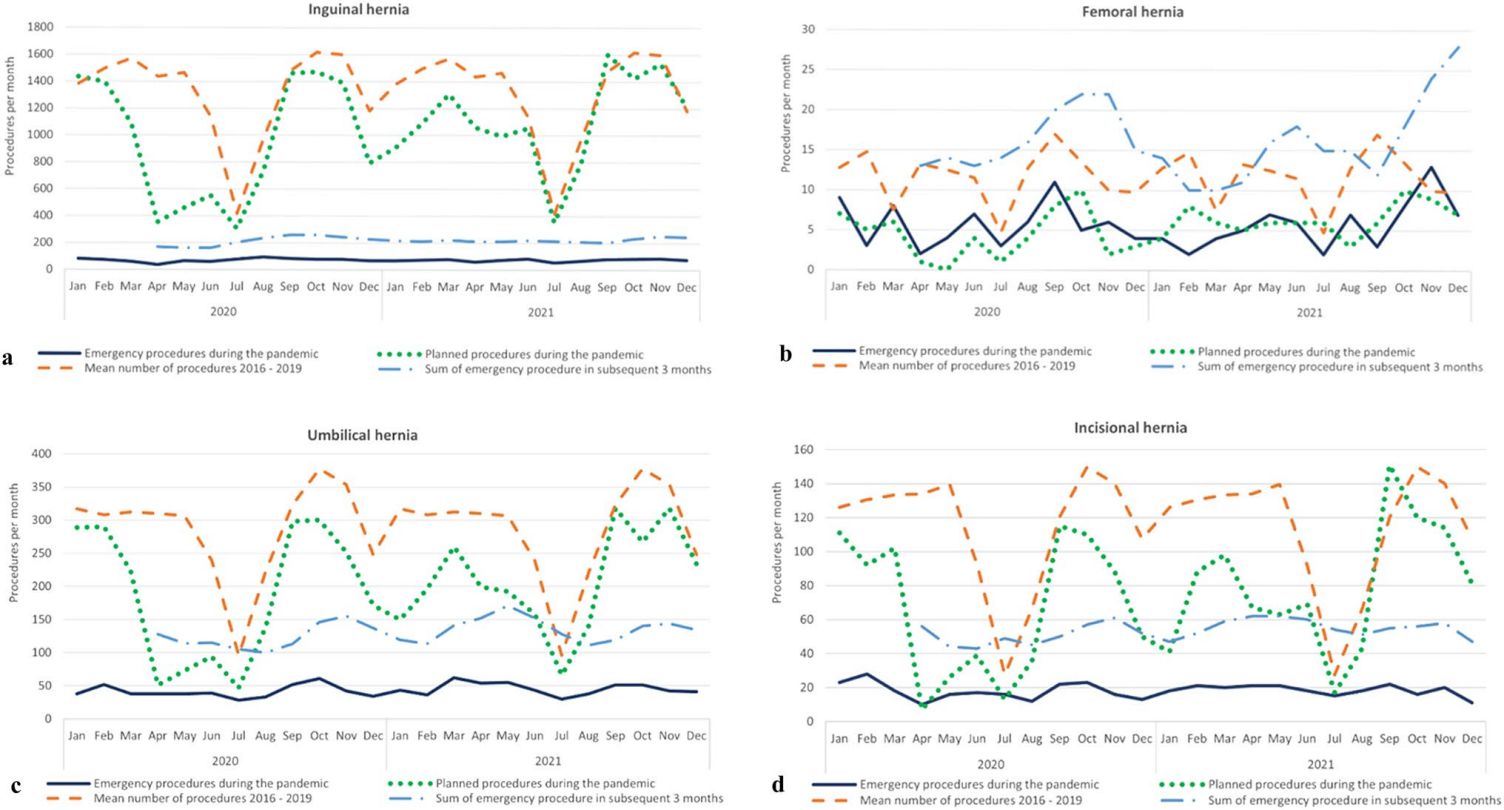
Numbers of hernia repairs during 2016–2021 for each hernia type (**a–d**). The COVID study period was 2020–2021, and the pre-pandemic period was 2016–2019

A total of 34,737 hernia repairs were identified during the COVID-19 study period. Of those, 31 373 (90%) were planned hernia repairs, and 3 364 (10%) were emergency repairs. During the pre-pandemic period from 2016 to 2019, a total of 41,357 planned hernia repairs were identified. Baseline data are presented in Table 1. Figure 2 presents an overview of the number of hernia repairs for each month. The numbers according to hernia type repaired each month are presented in Fig. 3.

**Table 1:** Baseline data for hernia repairs from January 1, 2021 to December 31, 2021

	Pre-pandemic period (2016–2019)	COVID study period (2020–2021)
Mean age, years (SD)	57 (21)	59 (20)
Men	96 863 (82%)	28 941 (83%)
Women	20 588 (18%)	5 800 (17%)
Planned inguinal hernia repairs	84 168 (94%)	24 782 (93%)
Emergency inguinal hernia repairs	5403 (6%)	1754 (7%)
All inguinal hernia repairs	89 571	26 536
Planned femoral hernia repairs	457 (56%)	127 (48%)
Emergency femoral hernia repairs	366 (44%)	136 (52%)
All femoral hernia repairs	823	263
Planned umbilical hernia repairs	16 234 (84%)	4 723 (82%)
Emergency umbilical hernia repairs	3175 (16%)	1 039 (18%)
All umbilical hernia repairs	19 409	5 762
Planned incisional hernia repairs	6215 (81%)	1 745 (80%)
Emergency incisional hernia repairs	1433 (19%)	435 (20%)
All incisional hernia repairs	7648	2 180

No correlation was observed between the number of elective repairs performed each month and the number of emergency repairs during the following three months for any hernia type. Pearson correlation coefficients were − 0.364 (*p* = 0.114) for inguinal hernia, − 0.042 (*p* = 0.859) for femoral hernia, 0.044 (*p* = 0.855) for umbilical hernia, and − 0.303 (*p* = 0.193) for incisional hernia repairs.

## Discussion

The COVID-19 pandemic had a great impact on hernia surgeries in Sweden, especially during the first wave. However, there were no statistically significant correlations between the number of elective hernia repairs and the number of emergency repairs in the subsequent 3 months during the COVID-19 study period.

A retrospective study by Ewing et al. [17] assessed the impact of fewer benign elective hernia repairs on the number of emergency hernia repairs during the COVID-19 pandemic in Southeast Scotland. The authors concluded that there was a 24% increase in emergency inguinal hernia repairs during the COVID-19 pandemic lockdown despite the overall reduction in emergency hernia repairs [17]. Other studies based in Sweden [9] and Turkey [17] either showed no changes in the number of emergency inguinal repairs or found a decrease of emergency inguinal repairs during the COVID-19 period respectively. However, both aforementioned studies found that the number of emergency ventral hernia repairs increased during the COVID-19 pandemic period. Nevertheless, despite an increase in emergency ventral hernia repairs, the reduction in numbers of overall benign hernia repairs did not result in a higher risk for strangulation [9].

The risk of incarceration or other emergency events in cases of postponement of planned procedures is difficult to predict, as the natural course of hernias is not fully understood [19]. In two randomized pre-pandemic trials among men with groin hernias with few or minor symptoms, the risk of emergency events was found to be low in the watchful waiting groups [18, 21]. Watchful waiting for asymptomatic groin hernias has been found to be safe and cost-effective among patients who are under 50 years of age with an inguinal hernia with a duration of signs longer than 3 months [22].

Whereas there is evidence in support of watchful waiting regarding the outcome of inguinal hernias, less is known regarding femoral and incisional hernias. During the early phase of the pandemic, it was feared that cancelation of elective surgery for hernias assumed to be at high risk of incarceration would lead to increased numbers of patients admitted for incarcerated hernias, especially incisional and femoral hernias. However, in the present survey, only a minor increase in emergency events was seen during the COVID period. Most femoral hernias presenting with incarceration are seen in women without previous history of a diagnosed femoral hernia [19]. Although we cannot confirm this in the present study, we believe that the natural course of a groin hernias in women is not the same as that of an occult femoral hernia.

The risk of incarceration occurs in the first three months after symptom presentation [24]. Nevertheless, no dramatic increase in emergency procedures was seen during the pandemic in the present cohort. Most planned procedures are carried out more than three months after the first symptom, and it is thus difficult to assess the impact of planned procedures on the risk of acute strangulation.

The present study reflected outcomes at the population level. There may have been patients awaiting an elective procedure who eventually developed incarceration that was either reduced with or without contact with a surgeon. Some patients may have refrained from seeking surgical care during the pandemic, either by reducing their incarcerated hernia themselves or by seeking help outside the emergency care unit. There may also have been patients with an irreducible hernia with a fatal outcome who did not undergo emergency surgery. More studies are needed to predict the natural course of hernias managed with watchful waiting that stratify by age, sex, and type of hernia. Postponing a hernia repair may limit the patient’s ability to perform daily activities, even if the hernia does not require emergency repair. The timeliness of hernia repair remains an important issue.

This study was limited by the validity of the National Patient Register. The National Patient Register has national coverage of inpatient as well as outpatient procedures. There may, however, have been procedures inaccurately classified regarding hernia type. There is also a lack of uniform criteria for emergency repair. This group may have included repairs carried out shortly after an incarcerated hernia had been reduced or on patients with an irreducible hernia. We also lacked data on hernia strangulation.

In conclusion, COVID-19 has had a considerable impact on planned hernia surgeries. It is difficult to judge how the postponement of planned procedures has affected quality of life or has limited the function of those whose procedures were postponed. However, the cancelation of elective procedures did not lead to a substantial increase in emergency repairs. More studies are needed to fully understand the natural course of patients with a femoral, incisional, or umbilical hernia managed with watchful waiting.

## Data Availability

(Not applicable).

## Code availability

(Not applicable).
